# "Maybe we should talk about it anyway": a qualitative study of understanding expectations and use of an established technology innovation in caring practices

**DOI:** 10.1186/s12913-017-2587-3

**Published:** 2017-09-15

**Authors:** Randi Stokke

**Affiliations:** 1 NTNU Norwegian University of Science and Technology, Centre for Care Research, P.O. Box 191, 2802 Gjøvik, Norway; 2 Inland Norway University of Applied Sciences - INN University, The Centre for Innovation in Services, P.O. Box 400, 2418 Elverum, Norway

**Keywords:** Home care, Welfare technology, Telecare, Social alarm, Personal Emergency Response System (PERS), Older people, Script, Domestication, Innovation

## Abstract

**Background:**

Technological innovations are strongly promoted to meet the demands posed by increased pressure on home care services and to assist ageing in place in western societies. Although heavily advocated as plug and play solutions, technologies have proven difficult and unpredictable when integrated into home care services. We need greater insight into what happens when technologies are integrated into caring practices. All technologies come with expectations as to their function. This study explores how actors who are involved with the social alarm, which is an established technology innovation, relate to, perceive and articulate these expectations of the technology in everyday living.

**Methods:**

The article presents results from a two-case study, using a triangulation of qualitative methods in order to gain an in-depth understanding of technology in use in home care services through “thick descriptions”. The study was conducted in Norway and data were analysed using a stepwise deductive-inductive analysis.

**Results:**

The empirical findings demonstrate that expectations regarding the social alarm, even though it represents a simple and well-established technology, are complex and multidimensional. The notion of script and domestication provided relevant tools for exploring these expectations and for understanding how actors interpret and adapt their practices of using the technology. This enabled a more comprehensive understanding of how technology opens up for different interpretations and puts values in play.

**Conclusions:**

This article suggests exploring technology in use as scripted in multidimensional script, and offers a frame for doing so. It also reveals how technology scripts and articulation prove important for understanding the complex reality when integrated into home care practices, thus identifying how using the technology leads to the taming and unleashing of both technology and actors. The study offers an increased understanding of how and why technology is unpredictable and works differently in different contexts. Moreover, it stresses the importance of avoiding expectations of plug-and-play in a reality of complex interactions between different actors.

## Background

In most societies, there is increased pressure on and a growing demand for home care services [1] due to demographic ageing, economic pressure and changes in policy in terms of prioritising independent living [2–4]. This pressure has led to increased focus on telecare innovations for the home care services as a promising solution for tackling the challenges [5, 6].

### Technology in caring practices

The term “telecare” has different implications [7]. However, in this context it embraces different kinds of technical devices and services with integrated alarms, sensors and other equipment aiming to help fragile, dependent people live independently [7–9]. A range of promises accompany the use of telecare in care practices as well as expectations such as contributing towards active ageing, preventing/delaying admission to long-term care institutions and reducing caring costs [1, 10, 11].

Advocates tend to describe telecare as “plug-n-play” solutions, providing effective help by placement of devices at home [12]. However, there seems to be a gap between the anticipated use and actual practice of telecare, as the integration and adoption have proven difficult. Many projects never get beyond the pilot stage [13–15] and implementation is on a smaller scale than expected. Likewise, telecare tends to be more complex than policy promises suggest [16]. Little attention has been paid to the social constructs in which telecare use actually takes place [6, 15, 17].

There is a need to shift our focus and think differently around telecare in use. Instead of viewing technology as a black box implemented into an existing reality, we need to acknowledge that technology is an integrated part of care practice. Telecare implies a different kind of care with complex interactions between multiple actors and a wide variety of technology and changing roles, thus redefining how actors live, work, and even identify their lives [18]. J Pols [12] describes how telecare contributes to create different kinds of care entailing different problems and solutions. We thereby change the research focus from the assumed effects of technology innovations, towards viewing technology, the human actors involved and society as heterogeneous networks [19, 20].

In addition, we tend to expect the same effect from different kinds of technologies in different caring contexts. However, this is rather problematic, as we often end up treating different technologies and contexts in the same way. Instead, we need to analyse what happens in a specific context with specific types of technologies, to explore who might benefit from the technology and what kind of practices they participate in creating [21].

This article explores an established telecare integrated into home care practice, how actors relate to the expectations that come with the technology and how this affects actors’ interpretation of and engagement in the technology. It was fruitful to choose an established technology since this opened up for studying the emergence of personal, professional and organisational issues that have not yet surfaced in new technology innovations.

### The social alarm

The empirical focus of this article is the social alarm, the most common telecare solution in use in caring practices in western societies [22]. The social alarm is a technological device and an integrated service. The device consists of a unit placed centrally in the home. A pendant, a necklace/wristband with a button that the user can press when in need, allows open communication between the user and a dedicated responder, enabling proper response. The range of the pendant is normally inside the house and partly in the garden. The alarm has the potential to incorporate a range of devices [23–25], and is often the starting point for additional alarms and applications. How the social alarm service is organised differs – from private arrangements where the alarm goes to a nominated contact (often a daughter), to smaller or larger public or private call centres answering and effecting the proper response [17, 24, 26]. The social alarm is integrated into a variety of contexts [12], and its organisation and use are part of different integrated caring practices. Although the social alarm is a well-established technology for use in home care services for older people, there are contradictory research results that describe a variety of practices and user experiences [17]. When taking context and use into account, the social alarm cannot be viewed as one singular entity with predictable characteristics, but rather a variety of practices with different interactions between the actors involved [11]. Examples of actors involved in the use of the alarm are the end user, relatives, neighbours, home care nurses, and telecare facilitators, who all have different expectations, experiences, roles, and relationships with each other and the technology. The end user is usually an older person with the alarm in his/her home [17, 27, 28].

### Theoretical framework

To describe and understand the anticipations of users, and what really goes on in the encounter between human actors and technologies, the notion of script and domestication may prove fruitful. Scripts and domestication provide us with approaches that render possible in-depth insights into a reality that otherwise is difficult to articulate and focus.

#### The script of technology

Designers and technologists seem to envision a representation of how a technology will function when designing a technology, imagining users’ needs and relation to the technology [29–32]*.* M Akrich [31] describes how this may be analysed by using scripts as a metaphor. A movie or a play contains a script, prescribing roles and relations between different actors (human and non-human), enacting and making manifest a specific normativity, and thereby shaping the world the actors live in. In the same way, the designers’ and technologists’ representation of the users’ behaviour materialises into a script with expectations of how the actors involved with the alarm will interpret, adapt and relate to the technology.

A technology can be designed with a strong or weak inscription. A strong script gives a firm and strong direction for the use of the artefact or device, while a weak script opens up for more flexibility in the use of the technology [33]. In this way the script indicates the necessity of the technology and its flexibility in the meeting with its users. A technology’s script does not determine its actual use and the script will be interpreted (descripted) in practice by the users of the technology. In fact, some users are rather creative in finding alternative ways to use technology other than the one intended [5], or even reject the script altogether (antiprogram). M Akrich [31] describes how it is necessary to go back and forth between the technology and the actors, and follow the negotiations between them to understand what happens.

Technology in practice is never completely fixed – there is always an interpretative flexibility. This means that the technology as an artefact opens up for different interpretations of how to use it, what to think of it, what feelings the technology inspires in the users etc. [34, 35]. How open the technology is to different interpretations depends on how strongly it is scripted and how the actors domesticate the technology among other things. In this paper, the notion of domestication represents a way of describing these processes of negotiation or interaction when actors meet technology, and how actors create a sense of ownership of the technology. Thus, what is interesting is not the chimaera of what advocates of telecare promote, but rather what really happens when technology is used in caring practices.

#### Domesticating, taming and unleashing technology

The notion of domestication as described by R Silverstone [36, 37] inspired by the process of domesticating wild animals, offers us a way of understanding the process of how technical objects gradually become an integrated part of someone’s everyday life. Domesticated technologies usually have a recognisable repertoire of how they are normally used in a cultural context [5, 11]. The domestication process is relational, where different actors, as well as the technology, have a social impact [36, 38]. R Silverstone [36] describes this process through four overlapping dimensions of appropriations: *commodification* (also called acquisition [30, 39]), *objectification, incorporation* and *conversion*. He describes the entire process from acquiring an object, through a complex process of negotiations between the technologies, how the object is scripted, and human actors involved. He further explains how the technology gradually becomes a part of everyday living, describing the values, pride, resistance, refusal, and tension in the interaction between humans and the technology, and how it gradually changes from something strange to an integrated part of everyday life [30, 36].

J Pols [21] merges domestication theory and script theory when she describes the taming and unleashing of technology in caring practices that deliver four heuristics. She claims that this can provide tools for studying the interaction between actors and technologies in caring practices. The four heuristics are 1. Actors might tame the technologies by using them to pursue their goals, either by finding new ways of use, or exploiting only some of the possibilities the technology offers. 2. Technologies can unleash their users, making them request new services from the technology. 3. Technologies sometimes tame their users by making them somehow dependent on the technology. 4. Technologies can also unleash unexpected practices, leading to new areas of use [21].

The aim of this article is to contribute to ways of understanding the complex reality of telecare in use in caring practices as described earlier in this article. Further to develop the notion of the script as an analytical tool. Even though the notions of script and domestication are well established, these terms are relevant to use and develop further to increase our ability to understand the complexity and unpredictability of the use of telecare innovations in home care practices in the future.

The following research question will be addressed: How can exploring telecare scripts help us to understand different actors’ interpretation, integration and utilisation of telecare in home care services?

## Methods

### Design

A two-case study inspired by the principles of the ethnographic approach was conducted. Data for this study were collected in the period from August 2014 – December 2015. Using a variety of methods when studying complex phenomena opens up for more breadth and depth, and enables a more comprehensive picture of practice [40]. This involved a triangulation of qualitative methods in order to gain an in-depth understanding of the technology in use in the home care services through “thick descriptions”. “Thick descriptions” refer to accurately describing and interpreting observed, read or spoken social action related to a given context [41]. The context in this study is the different actors’ experiences with the social alarm in use in home care services. Moreover, we seek to capture in detail their thoughts and feelings and the complexity of the relationship between the actors, the technology and the service as described by JG Ponterotto [41]. This study combines participant observation, in-depth interviews, and the examination of relevant documents in two strategically-chosen municipalities in Norway where this study is conducted. In Norway, municipalities are the lowest administrative and political organizational level. There are 426 municipalities in Norway (May 2017) of different sizes and demography [42].

The content of municipal home care services varies within different countries – from functioning as a safety net for people without relatives to a right for all citizens [1]. In Norway, the home care services are responsible for assisting people in need by providing health and care services at home [1, 4, 43].

### Sample and settings

The municipalities were strategically chosen for maximum diversity, as they represented two typical and very different local communities in Norway. The first municipality is a mid-size city in the inland region with 30,000 inhabitants. The second municipality is in a rural location on an island in the north of Norway with 2600 inhabitants. The author had no previous relationship with the participants before data collection. Since older people are the main end users of the social alarm, this affected the strategic participant selection aiming for maximum diversity as described in Table 1. The end users lived either in their own home or in residential home care facilities. The latter were small flats, often located close to a nursing home. They are registered as private homes, and service needs are met by the home care services. People living there are usually dependent and frail.

**Table 1:** The criteria for strategic recruitment

Participants	Recruiting criteria. Aiming for maximum diversity
End users	Had possessed the social alarm for more than one year. Different experiences with activating the alarm. Both sexes, a variety of age, living conditions and dependency.
Next of kin	Next of kin with different relationship to the end user. Difference in interactions, and how far they lived from the end user.
Home Care workers.	Experience with the social alarm. Different background and work responsibility related to the social alarm.

The author conducted a one-week participant observation in the home care services in each municipality, to become familiar with the use of the social alarm in the various cultural contexts. This involved accompanying home care personnel responsible for receiving alarm calls on their visits to older people, while focusing on the use of the social alarm. Situations observed when accompanying the telecare-responsible employee in both municipalities in their work were the installation process, training of users and administration of the alarm service. Detailed notes of the sequence of action as well as the interaction between the actors were taken.

Documents related to the social alarm in the two municipalities were studied. These included information on the municipalities’ websites, written information material distributed to the end users during installation of the alarm and guidelines for the health care personnel who administered and handled activated alarms. The author conducted all empirical work and analysis.

The home care manager in each municipality recruited participants for interviews. The interviews were conducted with end users who had a social alarm (*n* = 11), relatives of people with the alarm (*n* = 7), and care-workers (*n* = 9), including key workers and managers (*n* = 3). Some of the respondents had several roles, e.g. both being a spouse and an end user, or a home care worker and a daughter. Twenty-two interviews were conducted using Critical Incidence Technique (CIT). CIT is a practically oriented, commonly used explorative approach that facilitates an understanding of the complexities of an event, and the interactions between different actors involved. CIT focuses on the respondent’s descriptions of specific incidents [44, 45] – in this study their experiences with the social alarm in use. This generates insights and uncovers a tacit understanding of an incident, including affective, cognitive and behavioural elements [46, 47]. CIT can be used within different research traditions. This study is within a phenomenological-interpretivist tradition, as developed by Elizabeth Chell. Thus, incidents are described as something that emerge from the practice and are embedded in the actors’ perspective [45, 46, 48].

The informants were asked to tell stories, both positive and negative, which reflected their experiences with the social alarm, but the author also tried to elicit stories by questions and follow-up. All interviews were conducted in the respondents’ home or at home care facilities, and typically lasted about one hour.

### Analysis

**Fig. 1:**
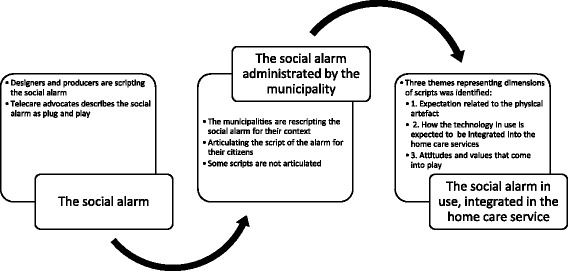
The scripting process of the social alarm that emerged in this study

All interviews were audio-recorded and fully transcribed verbatim. In accordance with CIT [44], the focus of the analysis was on the participants` perspectives. Detailed field notes from the observations and transcriptions from the interviews were analysed thematically by a stepwise deductive-inductive analysis [49]. First, the observation notes, the transcripts and the documents were analysed separately, starting with a detailed coding resulting in a total of 68 empirically closed codes focusing on the participants’ perspectives. The relationship between the codes from the interviews, observations and written material was examined. These codes were merged and condensed, and grouped into 11 categories. Three of the categories were relevant for this article: how the social alarm is scripted, how the scripts are articulated, and the taming and unleashing of the social alarm through domestication in caring practices. These categories were further explored, resulting in three broad themes related to expectations and use of the social alarm relevant for this article. This mainly inductive qualitative analysis was rooted in the actors’ perspectives, focusing on their “voices” [49]. The themes emerged from going back and forth between theory and empirical data using theory as a sensitising tool for the analysis. The themes that emerged formed the empirical-analytical basis of the article: 1. Expectations related to the physical artefact, 2. How the technology in use is expected to be integrated into the home care services, and 3. Attitudes and values that come into play. These themes embrace the process of interaction between the different actors involved and the technology, from the time the technology is presented until it is integrated or refused by the end user, as displayed in Fig. 1.

Some results were elucidated through written and oral information while others emerged through the actor’s experience and descriptions. The analysis provided a rich and extensive material, and in this article, short examples are given to provide empirical illustrations. NVIVO 11 software for qualitative data analysis was used as a tool when organising, analysing and finding insights in the material.

The following section presents the results of the analysis from the interviews, observations and written material related to expectations and use of the social alarm integrated into caring practices. The 11 end user respondents interviewed consisted of 4 males and 7 females, age 59–98. All end users had possessed the social alarm for many years. Most respondents did not remember for how long. All respondents were dependent and in need of assistance from the home care service. They all had one or more chronic diseases. The next of kin interviewed were either daughters (3), a friend (1), or a spouse (3). The home care workers were nurses (4), other care workers (5) and/or had the role as key workers and managers (3).

## Results

The results will first present how the municipalities included in this study re-scripted the social alarm. Furthermore, the paper will address the scripting the social alarm that emerged during the analysis, and how this is interpreted and lived out among the different users through domestication. Humans and technology are linked together relationally [31, 50], and any distinction between them should be purely analytical rather than ontological [51], thus arguing against technology determinism.

In Norway, the municipalities administer the social alarm, offering and allocating the alarm to the target group. Both municipalities in this study provide information about the social alarm on their websites. They offer the social alarm as a service for frail older people with disabilities who are living alone and might be in need of help, describing the service briefly. The primary home care services thereby inscribe their visions for the social alarm. The services are thus involved as an actor both in the use of the alarm and likewise re-scripting the alarm for use in the context. This is related to how the service providers view the alarm, and how they want the other actors to perceive it. Fig. 1 describes this scripting process, illustrating how scripting is an ongoing process. Three interrelated dimensions emerged from the data when exploring how the social alarm in these municipalities is scripted, as described in Fig. 1.

The paper will further explore how different actors interpret and relate to these three different dimensions, with illustrative quotations for each dimension.

### Expectations related to the physical artefact; how actors interpret and relate to them

The first dimension of how the social alarm is scripted that emerged from the analysis involves expectations of the cognitive and practical use of the physical device. This embraces the vision of the practical work of using the alarm as well as the cognitive work of understanding how to use it.

The end users acquired the social alarm, often at the suggestion of relatives or caregivers, or motivated by neighbours’ or friends’ positive experience with the alarm when in need. They describe how they acquired the alarm because they thought they needed it to feel safe living on their own, to get help when in need, or because the care workers or relatives wanted them to.

Observational notes describe how the caregiver responsible for the alarm provided oral and written instruction in the practical use of the device in both municipalities. End users were instructed in how to use the alarm when in need, what happened if they pressed the button, whom they would talk to, and who would come to their assistance.

The alarm device is strongly scripted and leaves little room for interpretative flexibility in practical use since there is only one button to press. “Jenny” stated during an interview that it was easy to learn and master the social alarm, even for people with little technological skills.

*“Jenny”: It is so easy to use. I just press the button on the pendant and then they answer….*

However, even though activating the alarm was easy, it was clear that the user needed sufficient cognitive capacity to remember to use it in such stressful situations. It was therefore not suited for people with cognitive impairment. “Anita”, a nurse working nightshifts told in an interview how end users omitted to activate the alarm.

*...so she fell backwards into her armchair and she was lying with her legs on top of the walking frame and her upper body in the armchair. Sounds strange, but she probably sat down on the walking frame and fell backwards and then ... And she lay there and got nowhere, and struggled and floundered about and was exhausted. She could not get up and did not activate the alarm pendant that she had around her neck….*

Both municipalities assessed end users’ needs and ability to utilise the alarm when a person applied for it. However, they had no guidelines or routines regarding what to do if the users no longer mastered the technology. They seldom withdrew the alarm unless the person died or was admitted to a nursing home. This topic was hardly ever a subject of discussion. Some care personnel mentioned during the observational study that it felt difficult to take away a service that people had been granted, even if the end user was unable to utilise the alarm any longer.

### How the technology in use is expected to be integrated into the home care services

The second dimension of the script that emerged is how the use of the social alarm is expected to be integrated into the home care services. This embraces the network of end users and care workers, and also includes neighbours, family, the rest of the care service and other parts of the municipality.

#### The integration of the alarm into the home care services in the two municipalities

When an end user activates the alarm, the alarm plays an active role within the home care services. This is organised differently in the two municipalities participating in this study as described in Table 2.

**Table 2:** How the social alarm in integrated into the home care services in the two municipalities

	Municipality 1	Municipality 2
Who answers the activated alarm	Home care nurse on duty	Private alarm call centre, with dedicated personal
Respondents’ profession	Health care worker, nurse	Not health care workers
Respondent knows the alarm activator	Often, since the alarm activator was a frequent user of home care service	Usually not. The alarm centre services many municipalities
Receiving the alarm	Home care nurse talked to the alarm activator if possible	The call centre personnel talked to the alarm activator if possible
Effectuating the response	The home care nurse either visited the person in need or activated other proper response	Most frequently, the call centre called the home care nurse and they visited the person in need or activated other proper response
Benefits of the organisation	Health care professional answered all alarm calls. The respondent usually knows the activator	Home care workers are only disturbed when necessary
Drawbacks of the organisation	Home care nurses are disturbed unnecessarily during their work by activated alarms that could have been checked out by other personnel (false alarms, requests etc.)	The personnel manning the call centre are not health care workers. They do not know the end user activating the alarm

In municipality one, the care-worker was usually helping other patients when they received an alarm. Due to confidentiality, they had to leave the room to talk and were therefore interrupted in their work. There are many field notes describing how the person receiving help commented on this, as he/she had to wait for the care-worker to return and continue the work. Answering the alarm call had priority in the care-workers’ duties since it could involve life-threatening emergencies. As described in Table 2, the benefit of this method of organisation was that those answering the alarm are care personnel and they often know the person activating the alarm. Therefore, they were able to respond effectively. Several of the end users mentioned that it felt safe knowing the person responding to the alarm.

The benefit of the method of organisation in the second municipality was that the call centre would answer all alarms so that the home care services would only be disturbed when necessary. Alarm calls not requiring attention from the home care worker never reached nor interrupted their work. However, since the alarm centre personnel were not health care personnel and did not know the person activating the alarm, this made it sometimes more challenging to know what would be the best response to an alarm. In both municipalities, the social alarm plays an active role in the practical work of the home care worker every time an activated alarm demanded the care workers’ attention.

#### Different actors’ description of the aim and purpose of the social alarm

The written information provided by both municipalities informs the end users that they should activate the alarm when in need, but neither municipality specified what counted as “in need”. In practice, there were many versions of the “truth” of what counted as legitimate alarm-activating purposes. The health care personnel had different opinions about legitimate reasons for activating the alarm as opposed to misuse. The purpose of the alarm seems to be unclear. Here is an excerpt from an interview with nurse “Anne”.

*... I’ve never really experienced any misuse of the alarm. The callers always have a reason. But it depends on what you consider to be misuse. ... some people think it’s misuse if you use it to call for help to get to bed. But it isn’t really because they’re not able to do that on their own. … Sometimes there have been maybe ... that patients have called mostly due to anxiety. But ... that’s not misuse either because they are not well. ... So I can’t really remember any misuse. They have a high threshold for activating the alarm ... so I rather think it is the other way around, that they don’t press the button when they really should have.*

Other respondents described in the interviews how they believed only extreme situations of a physical nature such as falling were legitimate emergencies. Several of the end users were reluctant to activate the alarm, fearing that their needs were not great enough. “Olga” reflected during an interview about her uncertainty as to whether her accidents were serious enough. She did not want to be a nuisance to the nurses; at the same time, she was often afraid and exhausted because of trying to get up after falls. Other end users said during the observational study that they could use the alarm whatever their need. Some had agreements with the care workers to use the alarm when they needed practical assistance, such as visiting the toilet or going to bed.

#### How the social alarm is integrated differently in different housing

In Norway, enabling older people to live independently in their private home rather than in nursing homes is a policy priority, even in the case of frail, dependent people. The end users in this study either lived in their original homes or had moved to residential facilities. The social alarm was used quite differently depending on these different living arrangements. Compared with people living in their original homes, those living in residential homes had a greater need of home care service in both municipalities. The end users living in residential homes used the alarm for other reasons and more frequently than those living in their original homes. Some end users used the alarm almost like pull cords are used in nursing homes. A number of people had acquired the social alarm before they moved to a residential home; others got it when they moved in. There were no differences in the expressed aim and purpose of the alarm related to the different living arrangements. However, there were fairly large differences in the interpretations of different actors about what was legitimate use of the alarm. “Maria” who had the alarm for several years before she moved to a residential home described it like this:

*I think that I and the other residents, we don’t agree on when you should use the alarm ... it is an alarm, and it should not be used other than when it is important, ... that you are injured or something like that. You should not use the alarm if you just need a little help with something. Certainly not. We don’t agree about this. I find that most people use the alarm too often.*

“Maria” felt the alarm was only for what she called real emergencies, and explained how other tenants in the residential homes used the alarm if they wanted help to get dressed or felt lonely.

This study reveals the various interpretations of the aim and purpose of the social alarm among the different respondents, especially in connection with the use of the alarm in different housing contexts. Most respondents could easily describe what they individually saw as the purpose of the alarm. However, in both municipalities, the scripts or rules of when to use the social alarm were not generally articulated. These were taken for granted, and hardly ever debated.

### Attitudes and values that come into play

The third recognised dimension concerns the scripts of attitudes and values that come into play for the different users regarding the social alarm in use. This relates to the different emotions, feelings and values related to the technology that are experienced by the different actors, and what actually happens.

Values presented in written and oral information included being able to stay at home longer, feeling safe, never feeling abandoned, having your privacy, and staying active and independent. At the same time, the (rather negative) value of being assessed as fragile, passive, anxious and dependent was also implied, since the information presented these qualities as characteristics of potential end users.

The social alarm was loaded with value for all respondents in this study, but the various actors experienced different values, which conflicted at times.

The scripts of the social alarm as being suitable for frail and dependent people affected some of the end users, who explained how they were initially reluctant to have the alarm, and hardly ever wore their pendant. As “Peter” mentioned at one of the home care visits:

*In the beginning, I must say… I thought it was disgusting to wear… I did not like it. I took it off at night and put it on the bedside table. However, I realised that was a really stupid thing to do.*

Respondents described how they wore the alarm pendant inside their clothing, if at all, not wanting to identify themselves or be identified as frail and dependent. This was observed many times during visits in the end users’ homes. “Peter” further explains how today, five years later, he uses the alarm all the time and feels it an integrated part of his everyday life. Others described how accidents or specific events changed their opinion of the alarm or how they had just got used to wearing it. Yet others never had any problem displaying the alarm, arguing that the alarm is “designed for people like us, and that is just how it is”.

Relatives and caregivers often emphasised during interviews the importance of the end user being safe and able to reach someone when in need. Both family members and care workers said that they worried about the wellbeing of their frail relatives and patients and found comfort in knowing that they could reach help by pressing the alarm button.

One of the aims and purposes of the social alarm is for the user to be able to maintain an active life and still feel safe at home. The social alarm helped end users to maintain an active life. For “Ola”, who lived in a two-storey building the alarm enabled him to take more chances and live more actively knowing he would be safe.

*It feels safe when I’m going downstairs to the basement….*

“Lisa” had the opposite experience. She described how she was warned about the range by a friend:

*She said to me: You must remember you cannot go far from the house… I only go out on to the steps.*

Some respondents described how they ended up living more passively due to anxiety that the pendant would not work if they went too far from the main unit.

Regardless of their reasons, several of the end users described experiencing conflict regarding the pros and cons of using the social alarm, especially when values come into play as previously described.

**Fig. 2:**
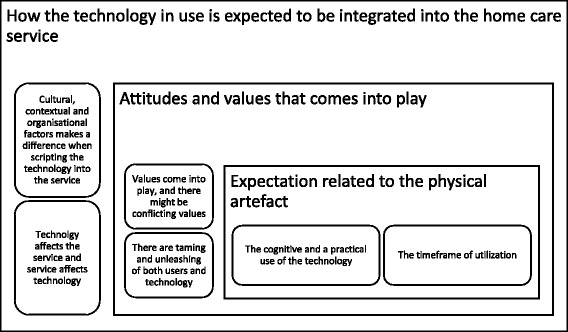
Presents an overview of the different dimensions of scripts that must be accounted for, and the themes that emerge when actors relate to the different scripts

Figure 2 presents the three interrelated dimensions of the script and domestication of the social alarm identified in this study that provide analytical tools. The Figure displays different factors that emerged when the scripts of the social alarm were interpreted, and the alarm interacted with the actors through the domestication process. This article will further discuss the factors presented in Fig. 2, with particular focus on those that are unarticulated since they may serve as indicators for some of the unpredictability of the alarm.

## Discussion

The aim of this paper is to increase our understanding of what happens when telecare is used in caring practices, and how these practices come about. Furthermore, focus is on developing the notion of the script as an analytical tool in order to highlight the complex reality of how actors interpret and relate to the expectations that come with the technology, and how this affects its utilisation and their engagement. As the results presented in Fig. 1 show, the different scripts of a technology are not just embedded into the technology by the designers and producers. They are in fact, descripted and rescripted into a different context. The municipalities rescripted the alarm as described in the results to represent the service they want to offer their citizens. This illustrates how even a simple and established technology may be rescripted to fit the different contexts in which it is integrated. Therefore, once the context is taken into account, the technology is no longer integrated into one practice, but rather a range of practices with different entities and challenges.

### The cognitive and practical use of technology and the timeframe of utilization

The alarm is scripted as easy to use and suitable for people with little technological skills. Indeed, the end users described how they felt safe having the social alarm and that it was easy to use. However, as the end users are ageing, they often become frailer and their health deteriorates. There seems to be a limit in time for how long most end users were able to activate the alarm in cognitively demanding and stressful situations. This utilisation timeframe is also seen in studies of other kinds of telecare in use, especially GPS tracking of people with dementia, where there is a window of time in which the end user is able to utilise the technology, and therefore the different care arrangements are temporary [52]. Technologies that are thought to be beneficial and to consolidate safety, might, in fact, be harmful to the patient if the patient is no longer able to get help when in need, as demonstrated by the social alarm. When this happens, the technology that used to be domesticated is no longer so, thus, leading to a rescripting, and a new domestication or no domestication at all.

### How we choose to organise the services in which the technology is an integrated part

Caring practices must be considered as situated solutions rather than a universal solution [53]. We need to widen the focus from focusing purely on the practical use of a technological artefact to focus also on the integrated context. The social alarm is an integrated part of different networks, including the service organisation. The different actors involved coordinate and organise their efforts in relation to the technology and other parts of the service. As presented in Fig. 2, cultural, contextual and organisational factors became apparent when integrating the alarm in home care practices. The end users expect to get help when activating the alarm, and they do. However, activating the alarm impacts the service in different ways since the care workers must reorganise their work, other patients have to wait, and the management has to take the alarm into consideration when planning the workflow for the home care services etc. In addition, the service is integrated with other services forming interrelated networks of actors and services that mutually reinforce each other. As described in the results, the social alarm is administered differently in the two municipalities so that the organization of work for the home care personnel is dissimilar, creating different practices. A nurse answering the alarm call has other work responsibilities as well, and every time the alarm is activated, it affects other patients and the care workers’ day.

### What scripts are we addressing

As mentioned above, this research revealed that the dimensions of scripting that emerged are only partially described and articulated regarding how the social alarm is integrated into the home care services. In fact, issues that were not addressed affected the use of the technology just as much as the scripts that were articulated by taming and unleashing the users in rather unpredictable ways. Three prominent example of this emerged. These are issues related to the range of the pendant, the living arrangements of the end users, and what counts as legitimate reasons for activating the alarm.

#### The range of the alarm pendant

As presented in the results there was uncertainty among the actors regarding the range of the alarm pendant. JC Aceros, J Pols and M Domènech [54] found that the script of the social alarm indicated an expectation of people staying at home, which was in opposition to the goal of active ageing. This study identified the same challenge, but also the opposite – that people felt safer and therefore became more active. Some users took chances even though they were unsure about the range of the pendant, and thereby tamed the technology, while others were tamed by the technology and chose to stay inside the house to be sure. Pols [21] describes the latter as taming of actors. It seems important to clarify the technological specifications to increase safety among the users and enable active ageing.

#### The living arrangements

The results show that in this study, living arrangements mattered even more than how the service was organised. Many older people move to residential facilities when they become more dependent. Although the articulated scripts of the social alarm are the same for both kinds of living conditions, they were differently interpreted. This led to differences in the practical use of the alarm, which again led to a different domestication for the users involved, and different caring practices. N Oudshoorn [55] found in her study that “place matters in shaping user-technology relations”, and that there is a “place-dependency of user-technology relations”. The same technology can mean different things in different contexts or housing and she argues that this is decisive for the meaning and purpose of the technology [55]. In this study, the municipalities described the same purpose for the alarm regardless of housing, but end users living in residential homes activated the alarm for other reasons and far more frequently than those living in their own homes, as they had other needs for help. This led to uncertainty among some users about what emergencies were sufficiently serious, indicating that they had been tamed by the technology since they ended up not activating the alarm even when in need. The social alarm as a technology has its functionality and necessity that affects the possible utilisation when technology meets expectations. However, other end users were able to tame the technology and found new reasons for activating the alarm in collaboration with the care personnel.

#### What counts as legitimate reasons for activating the alarm

Regarding what counts as legitimate reasons for activating the alarm, the scripts in both municipalities addressed the practical use of the technology. However, norms and aims for when to use the alarm were scarcely articulated and hardly ever discussed or addressed. As the results demonstrate, there was a divergence in the perception of what counted as legitimate reasons for activating the alarm, leading to uncertainty about when to use it. These both tamed and unleashed practices. Some end users were tamed by the technology, being afraid of activating the alarm. Others tamed the technology by activating the alarm for what some considered illegitimate use, e.g. using the alarm when they felt lonely or stressed. M Mort, C Roberts and B Callén [56] found that alternative use of the alarm, for instance when the user wanted social contact, was strongly discouraged. They also found that what counts as “proper” use varies within different contexts, as demonstrated by the results of this study. Not articulating and addressing the scripts and expectations of the purpose of the alarm might lead to increased uncertainty among the actors. The social alarm, being an established and simple technology, seems to be taken for granted, and the script is expected to be clear and the practice is based on this, though not articulated. On the other hand, it is never possible to articulate and address all aspects of a practice. The home care personnel only pay short visits to the end users of the alarm. In fact, in this study many of the end users do not have regular visits from the home care personnel at all, so the purpose of the social alarm could never be thoroughly debated. In addition, some sides of a practice will always be tacit. Even if one were able to discuss all sides of the scripting of a technology, there will always be an interpretative flexibility, making the technology unpredictable in use.

### Individual differences that we can never predict

We can never completely predict whether the actors involved in using technology in a caring practice will tame or unleash the technology, or whether the technology will tame and unleash the actors involved. This is partly because the context is complex, but also due to interpretative flexibility. The end users in this study interpret the scripts of the alarm quite differently even when they were in the same municipality and in the same kind of housing. This is clearly shown in the example regarding the range of the pendant presented above.

By focusing on the scripts as multiple rather than singular, we can identify the different dimensions and elucidate the expectations that come with the technology in use. It is possible to focus attention on the differences in the various actors’ interpretation and how this affects the service and the individual actors. The results of this study show how users of the alarm domesticate the alarm differently. Domestication will always be characterised by interpretative flexibility as well as relational networks the users are integrated in. In fact, it seems that the supposedly domestication of the technology embodied by the social alarm often never occurs because the script is unclear and not addressed within the context and society it is integrated.

### Attitudes and values that come into play

Even though the social alarm is an established technology and users are highly satisfied with it [17], different values related to the script of the alarm come into play when it is put to use. Stigma is a known factor for non-use of telecare technologies [7, 57]. The results describes how some respondents struggle to identify themselves as within the target group, and how they needed time to get used to the idea of the alarm. This is in accordance with what JC Aceros, J Pols and M Domènech [54] found: that users were reluctant to wear the alarm pendant because it forced them to identify themselves as more fragile and dependent than they felt.

Some users chose not to use the technology at all, or in quite a different way than the one intended. In line with the findings of Peine and Neven in their 2011 article, these results indicate that the domestication process is not just an adaption process to the technology, it is also a process where the users actively adapt the technology to fit in their personal context [5]. The end users tame the technology to suit their needs as described by J Pols [21].

## Conclusion

We can expect an increased focus on technology innovations in caring practices in the future, since the pressure on the primary health care service is likely to continue to increase due to demographic and policy changes. This article contributes to a better understanding of the challenges we experience when using technology in caring practices. Even simple established technologies like the social alarm meet challenges when integrated into the network of actors and practices that form the home care services. The technology, in fact, contributes to creating new caring practices, with new challenges. This emphasises that expectations of technologies as problem-free, off-the-shelf package innovations are unrealistic in the context of complex interactions in the care services. Instead, we should explore the complex reality we find in caring practices, as is the case here.

This paper shows how rather than searching for unattainable predictors for successful technology innovations in caring practices, we need to explore how technologies and human actors interact by taming and unleashing each other in contextualised practices. We must acknowledge that there are different end users, different devices, different practices and different contexts.

This article contributes theoretically by identifying how the notion of the script contains several scripting dimensions, thereby providing an analytical instrument. Figure 2 can serve as a tool when implementing telecare innovations in home care practices and contribute to an understanding that the script will always be multidimensional. In addition to the practical and cognitive use of a technology, Fig. 2 also allows us to focus on how the technology is integrated into service practices and indeed creates new practices. In addition, there will always be different values that come into play, and these may conflict. However, it is important to be aware that other themes may emerge within the different dimensions of the script than those found in this study. Moreover, focusing on individual themes carries the risk of leaving others unattended.

The article also shows that it is important to address the articulation of the scripts of even established and mundane technologies. The actors involved in the social alarm need to address the expectations of the alarm and the opportunities and challenges the technology creates for them. By doing so, the different stakeholders can optimise the use, and maximise the benefit of having the technology. This study shows how established, highly valued and simple technologies like the social alarm can make us forget to elucidate and discuss the aim and purpose of the technology. This leads not only to different users interpreting the alarm in accordance with their beliefs, but also to insecurity among others.

This study opens up for a more comprehensive understanding of how implicit scripts affect the description of technology in unpredictable ways. Identifying and articulating scripts that occur when technology innovations are integrated into different caring practices is necessary for understanding the complexity of interactions when integrating new technology innovations in care services. In addition, it is important to focus on articulating the script in relation to different contexts since the purpose and practical use differ between contexts.

One of the most important findings in this study is that the social alarm is scripted as plug and play, but that there is in fact a considerable degree of rescripting both within the service, living situations and different end users’ changing health and caring needs. This indicates the importance of investigating technology innovations further in contrast to today’s heavy focus on the implementing and piloting phase.

## Abbreviations

CIT: Critical Incidence Technique
PERS: Personal Emergency Response System
